# Evaluating the impact of testing strategies for the detection of nosocomial COVID-19 in English hospitals through data-driven modeling

**DOI:** 10.3389/fmed.2023.1166074

**Published:** 2023-10-11

**Authors:** Stephanie Evans, James Stimson, Diane Pople, Mark H. Wilcox, Russell Hope, Julie V. Robotham

**Affiliations:** ^1^HCAI, Fungal, AMR, AMU and Sepsis Division, UK Health Security Agency, London, United Kingdom; ^2^Statistics, Modelling and Economics, UK Health Security Agency, London, United Kingdom; ^3^NIHR Health Protection Research Unit in Modelling and Health Economics at Imperial College London in Partnership With UKHSA and the London School of Hygiene and Tropical Medicine, London, United Kingdom; ^4^Healthcare-Associated Infections Research Group, Leeds Institute of Medical Research, University of Leeds, Leeds, United Kingdom; ^5^Microbiology, Leeds Teaching Hospitals, Leeds, United Kingdom; ^6^NIHR Health Protection Research Unit in Healthcare-Associated Infections and Antimicrobial Resistance at University of Oxford in Partnership with UKHSA, Oxford, United Kingdom

**Keywords:** hospital-associated (or hospital-acquired) infection, COVID-19, nosocomial transmission, SARS-CoV-2, modeling, testing

## Abstract

**Introduction:**

During the first wave of the COVID-19 pandemic 293,204 inpatients in England tested positive for SARS-CoV-2. It is estimated that 1% of these cases were hospital-associated using European centre for disease prevention and control (ECDC) and Public Health England (PHE) definitions. Guidelines for preventing the spread of SARS-CoV-2 in hospitals have developed over time but the effectiveness and efficiency of testing strategies for preventing nosocomial transmission has not been explored.

**Methods:**

Using an individual-based model, parameterised using multiple datasets, we simulated the transmission of SARS-CoV-2 to patients and healthcare workers between March and August 2020 and evaluated the efficacy of different testing strategies. These strategies were: 0) Testing only symptomatic patients on admission; 1) Testing all patients on admission; 2) Testing all patients on admission and again between days 5 and 7, and 3) Testing all patients on admission, and again at days 3, and 5-7. In addition to admissions testing, patients that develop a symptomatic infection while in hospital were tested under all strategies. We evaluated the impact of testing strategy, test characteristics and hospital-related factors on the number of nosocomial patient infections.

**Results:**

Modelling suggests that 84.6% (95% CI: 84.3, 84.7) of community-acquired and 40.8% (40.3, 41.3) of hospital-associated SARS-CoV-2 infections are detectable before a patient is discharged from hospital. Testing all patients on admission and retesting after 3 or 5 days increases the proportion of nosocomial cases detected by 9.2%. Adding discharge testing increases detection by a further 1.5% (relative increase). Increasing occupancy rates, number of beds per bay, or the proportion of admissions wrongly suspected of having COVID-19 on admission and therefore incorrectly cohorted with COVID-19 patients, increases the rate of nosocomial transmission. Over 30,000 patients in England could have been discharged while incubating a non-detected SARS-CoV-2 infection during the first wave of the COVID-19 pandemic, of which 3.3% could have been identified by discharge screening. There was no significant difference in the rates of nosocomial transmission between testing strategies or when the turnaround time of the test was increased.

**Discussion:**

This study provides insight into the efficacy of testing strategies in a period unbiased by vaccines and variants. The findings are relevant as testing programs for SARS-CoV-2 are scaled back, and possibly if a new vaccine escaping variant emerges.

## Introduction

Coronavirus disease 2019 (COVID-19) is a respiratory disease caused by the virus severe acute respiratory syndrome coronavirus-2 (SARS-CoV-2) that was first detected in China in December 2019 (1). Since then, there has been widespread community transmission of the virus across the world (2–4), and a global pandemic was declared by the World Health Organisation (WHO) in March 2020 (5, 6). Nosocomial (healthcare-associated) transmission of SARS-CoV-2 was identified early in the COVID-19 pandemic, and there is evidence of transmission occurring within and between populations of patients and healthcare workers (HCWs) in various settings (7–13). From 01 March 2020 to 31 August 2020, over 30,000 cases in England alone (1% of the estimated 3 million cases) were potentially attributable to nosocomial transmission among hospital inpatients (14).

Guidance and protocols for preventing the spread of SARS-CoV-2 in hospitals issued by the UK government have changed throughout the pandemic with the increase in testing capacity and the advent of rapid testing. A key theme for infection prevention and control (IPC) is the efficient detection and effective isolation of infected patients (15), and modeling studies have shown that creating effective cohorts of patients can reduce nosocomial transmission by up to 35% (16).

Evaluating the impact of interventions designed to inhibit nosocomial transmission of a virus using real-world observational data is complicated by the concurrent changes in confounding factors such as community prevalence, improvement in testing capacity/turnaround time, natural variability between hospitals and regions, and the lack of a control group for comparison (17). Computational models can provide a solution to these issues, as simulations of controlled scenarios can be performed where only specific variables are varied (18). Such models have been used previously to demonstrate the effect of IPC strategies on the number of nosocomial infections in hospitals for pathogens such as methicillin-resistant *Staphylococcus aureus* (MRSA) (19), *Clostridium difficile* (20), and vancomycin-resistant enterococci (21). Modeling during the COVID-19 pandemic has been vital for explaining and interpreting data and has been applied to estimate attributable mortality rates (22, 23), the impact of vaccination (24, 25), and the transmission of SARS-CoV-2 in countries across the world (26, 27). Furthermore, computational models of nosocomial transmission of SARS-CoV-2 have also been developed (28–33), and we have previously published an analysis using an individual-based model (IBM) of nosocomial transmission within and between patient and HCW populations in an English acute hospital setting (34).

In this article, we present an analysis of the impact of different testing strategies, test characteristics, and hospital-related factors on the number of nosocomial infections in hospitalized inpatients using our previously described individual-based model IBM (16). Furthermore, since patients with undetected nosocomial infections can go on to transmit them in the community post-discharge (35), we also explore the additional burden and detection benefit of testing all patients who were not previously known COVID-19 cases on discharge from the hospital. The overarching aim of this study is to evaluate the impact of testing strategies for detecting COVID-19 cases in hospitals.

## Methods

### Individual-based model

The IBM of within-hospital transmission is parameterised using multiple national datasets and literature and is calibrated to reproduce the number of SARS-CoV-2 infections in HCWs and patients in an average English hospital (34). A detailed model description can be found in the appendix of (34), but key features of the model are described here. Values and definitions for parameters (those italicized in this section) are provided in Supplementary Table 1.

#### Admission and cohorting

COVID-19 patients are admitted to the hospital at a rate per bed that is equal to the average number of COVID-19 admissions per bed on that date in the North West of England region, selected to represent an average admission rate across the country from the National Health Service England (NHSE) Situation Report dataset (36). A number of non-infected patients are also admitted. This number is less than or equal to the number of beds available under the occupancy level (the proportion of beds allowed to be occupied at a single timepoint) specified in the particular parameter set under study using the model parameter *beds available*, with a default value of 85%. The model parameter *non_covid_sympt_prob* defines the probability that non-infected patients exhibit symptoms resembling COVID-19. Therefore, they would be incorrectly cohorted with true COVID-19 patients (except in the case of rapid admissions testing, where the results are returned instantly, allowing this group to be cohorted with true negative patients). COVID-19 patients are prioritized for admission and can be admitted even when the occupancy level of the simulated hospital (defined by the *bedsAvailable* parameter) is exceeded, provided that the total number of beds in the hospital is not exceeded. On admission to the hospital, patients are tested under the appropriate strategy (see *Testing Strategies*) and assigned a space in a bay that has capacity based on their COVID-19 status (symptomatic–unconfirmed with a test positive, and non-symptomatic–unconfirmed with a test negative). Patients are correctly cohorted if the turnaround time of the test is zero, and they test positive on admission while being infected, or if they are symptomatic on admission and truly positive when the test turnaround time is greater than zero.

#### Transmission

Patients can transmit to other patients within their bay through direct transmission, with the rate determined by the value of parameter *bP2P* per infected patient per time step. They can also transmit indirectly to other patients in the hospital, with the rate determined by the value of parameter *bP2P_hosp*, where indirect transmission represents all possible routes of indirect transmission, e.g., with contaminated fomites or HCWs acting as vectors for droplet transmission. The model assumes that all patients are at the same risk of indirect patient exposure. Infected HCWs can transmit to patients that they are in contact with, with a rate determined by the value of parameter *bH2P*. HCWs outside the hospital can be infected in the community at the rate determined by the value of the parameter *commHCW*. Inside the hospital, they can be infected by coming into contact with an infected patient at a rate determined by the value of the parameter *bP2H* or an infected HCW at a rate determined by the value of the parameter *bH2H*.

#### Testing

Patients undergo PCR testing upon admission. The probability of testing is determined by the value of the parameter “testOnAdmProb_Inf” if they exhibit symptoms of a COVID-19 infection or have COVID-19-like symptoms upon admission, even if they are not confirmed cases. Conversely, if they do not show COVID-19-like symptoms, the probability is determined by the parameter “testOnAdmProb_Other.” Symptomatic inpatients are tested at the rate determined by the value of the parameter testInHospProb. An infected individual is classified as detectable when they are in the infected state of the susceptible, exposed, infected, and recovered (SEIR) model (and undetectable when they are in the E or R states) and will be detected with a probability determined by the value of parameter testSens, which is taken to be the sensitivity of a PCR test. Test results are returned after a predefined turnaround time (TAT), which is determined by the value of the parameter testPeriodSteps, and those patients that have a returned positive test result are considered confirmed cases, regardless of their true status. HCWs are assumed to self-isolate upon developing symptoms at the rate determined by the value of the parameter absentThroughSick_self and are then absent for 10 days.

### Scenarios

#### Baseline

Under the baseline scenario, all patients are tested on admission. The hospital has 85% occupancy and is divided into 6 bed bays. Tests have a 1 day turnaround and offer 95% sensitivity and 90% specificity. There is a 0.1 probability that a non-COVID-19 patient will show COVID-19-like symptoms upon admission. Under this scenario, we assume that the probability that an infected patient remains asymptomatic for the entirety of their infection is 16%, which is in line with estimates from Byambasuren et al. (37).

#### Testing strategies

We define the following testing strategies based on those that have been implemented in England throughout the pandemic (Supplementary Table 2): 0) testing only symptomatic patients on admission (as was policy prior to any COVID-19-related policy change); (1) Testing all patients on admission (38); (2) Testing all patients on admission and retesting patients that remain in hospital between 5 and 7 days post-admission (39); (3) Testing all patients on admission and retesting patients that remain in hospital on days 3 and 5–7 post-admission (39), with the addition of testing for symptomatic inpatients under all strategies. We also explore the impact of additional discharge testing for each strategy. Other than the explicit discharge testing strategy, patients are discharged when their simulated length of stay has expired. We do not model the impact of discharge testing on return to social care.

### Additional factors varied

In addition to the testing strategies described above, we adjusted a number of test and hospital-specific parameters to explore their effect on the proportion of patients that developed a nosocomial SARS-CoV-2 infection (Table 1).

**Table 1:** Parameter values used for scenario analysis.

**Parameter**		**Baseline**	**Scenarios considered**
Turnaround time (TAT)	Length of time taken for test results to be returned	1 day	0, 2, 3, 4 days
Beds per bay	Number of beds per hospital bay	6	4, 1
Occupancy	Maximum proportion of all beds occupied	85%	65, 75%
Non-COVID-19 symptomatic probability	Probability a patient is assumed to be a COVID-19 patient on admission and therefore incorrectly cohorted (TAT > 0 scenarios only)	10%	5, 20%
Sensitivity	The probability of a positive result given that an individual is infected and tested	95%	75, 85%

### Simulations

To reduce the impact of aleatory uncertainty, the parameter set for each scenario was replicated 50 times. In all simulations, there was a 10 day burn-in period to establish a stable hospital population before the virus was introduced, and the model then ran for 150 days, covering the time period from 03 March 2020 to 01 August 2020. The simulation is calibrated to ensure the number of detected nosocomial cases is within the range given by Bhattacharya et al. (14) [see (16) for variability between potential parameter sets].

### Data and model calibration

The model was calibrated to national-level admissions data from the UK Health Security Agency's Secondary Uses Service and Second-Generation Surveillance System datasets in combination with data from the SARS-CoV2 immunity and reinfection evaluation SIREN study (40) and a meta-analysis of HCW infection rates early in the pandemic as previously described (16). The definitions of community and nosocomial infections are taken from Public Health England (PHE) and European Centre for Disease Prevention and Control (ECDC) guidance, which classifies a case as community-acquired if a patient tests positive within 2 days of admission and hospital-associated (with varying degrees of certainty) if they test positive from day 3 (14, 41).

## Results

### Infection status of hospitalized or hospital-associated COVID-19 cases over time

**Figure 1:**
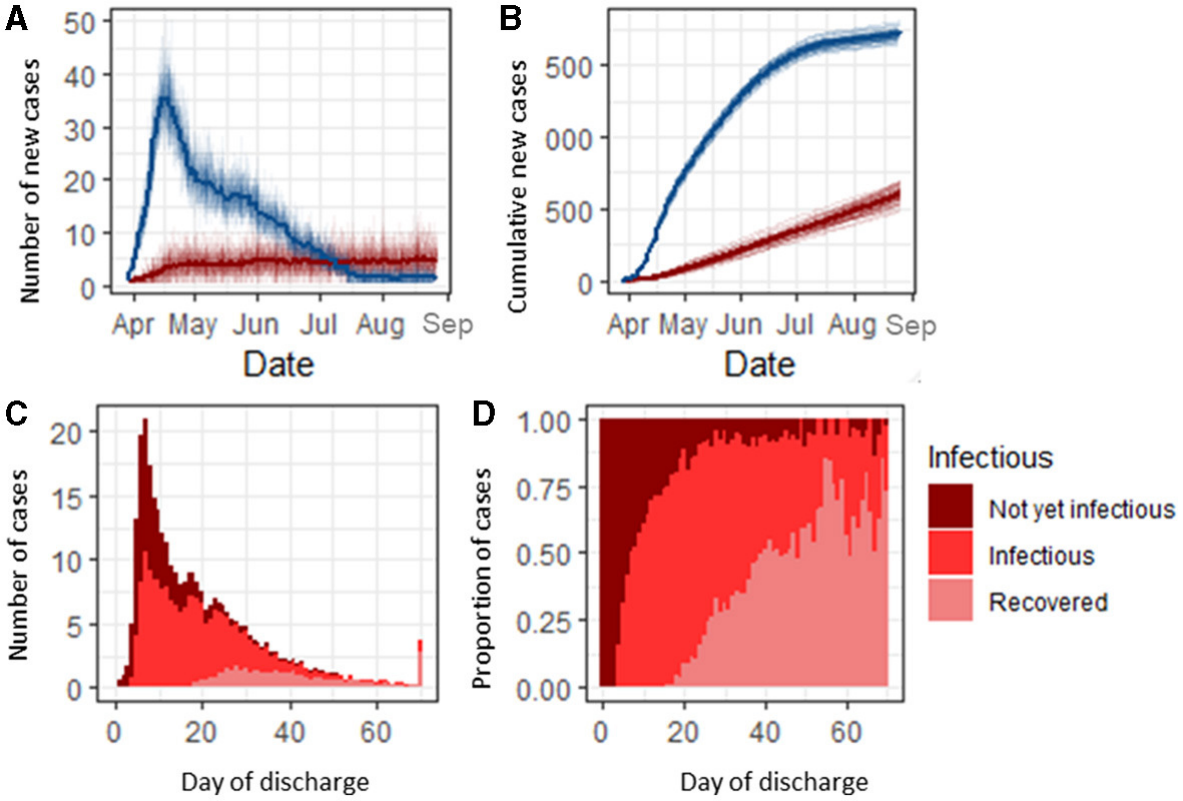
Classification and infection status of SARS-CoV-2-infected hospital inpatients. **(A)** Number of community-acquired SARS-CoV-2 admissions (blue) and nosocomial cases (red) per day. **(B)** Cumulative count of community-acquired admissions and nosocomial COVID-19 cases. **(C)** Infection status of infected nosocomial cases on the day of discharge. **(D)** Proportion of nosocomial cases discharged each day by status.

We simulated the dynamics of SARS-CoV-2 transmission in a typical English hospital (1,000 beds, 8,000 staff) between 03 March 2020 and 31 August 2020. The simulations followed the trajectories of both cases admitted from the general community and nosocomial cases (Figure 1A). During this time period, over 1,500 COVID-19 cases were admitted from the community, 77.5% (95% CI 77.4, 77.7) of which were infectious on admission (Supplementary Figure 1).

Under the baseline scenario, 1.6% (1.5, 1.7) of all susceptible admissions [318 (310, 325)] in our simulated 1,000 bed hospital developed a nosocomial infection over the 150 day simulation period (Figure 1B). Of the patients who developed a nosocomial infection, 46.6% (45.7, 47.5) were infectious before discharge (Figure 1C). Patients with a longer stay had a higher probability of being infectious before discharge, with those in hospital for longer than 20 days having an 81.7% (76.7, 86.9) chance of being infectious while in hospital (either currently infectious or recovered by the day of discharge). Nosocomially infected patients with a length of stay shorter than 5 days were unlikely [2.8%, (1.3, 4.4)] to be infectious in the hospital but progressed to becoming infectious post-discharge, and those with a length of stay between 5 and 25 days had up to a 65.9% (58.7, 73.1) probability of being infectious on discharge (Figure 1D). Before day 25, the majority of nosocomial cases were infectious on discharge and were either pre-symptomatic or asymptomatically infected (Supplementary Figure 2). Nosocomially infected inpatients were most likely to become infectious between 5 and 7 days into their stay (Supplementary Figure 1).

### Efficacy of testing strategies for detecting and confirming infections

**Figure 2:**
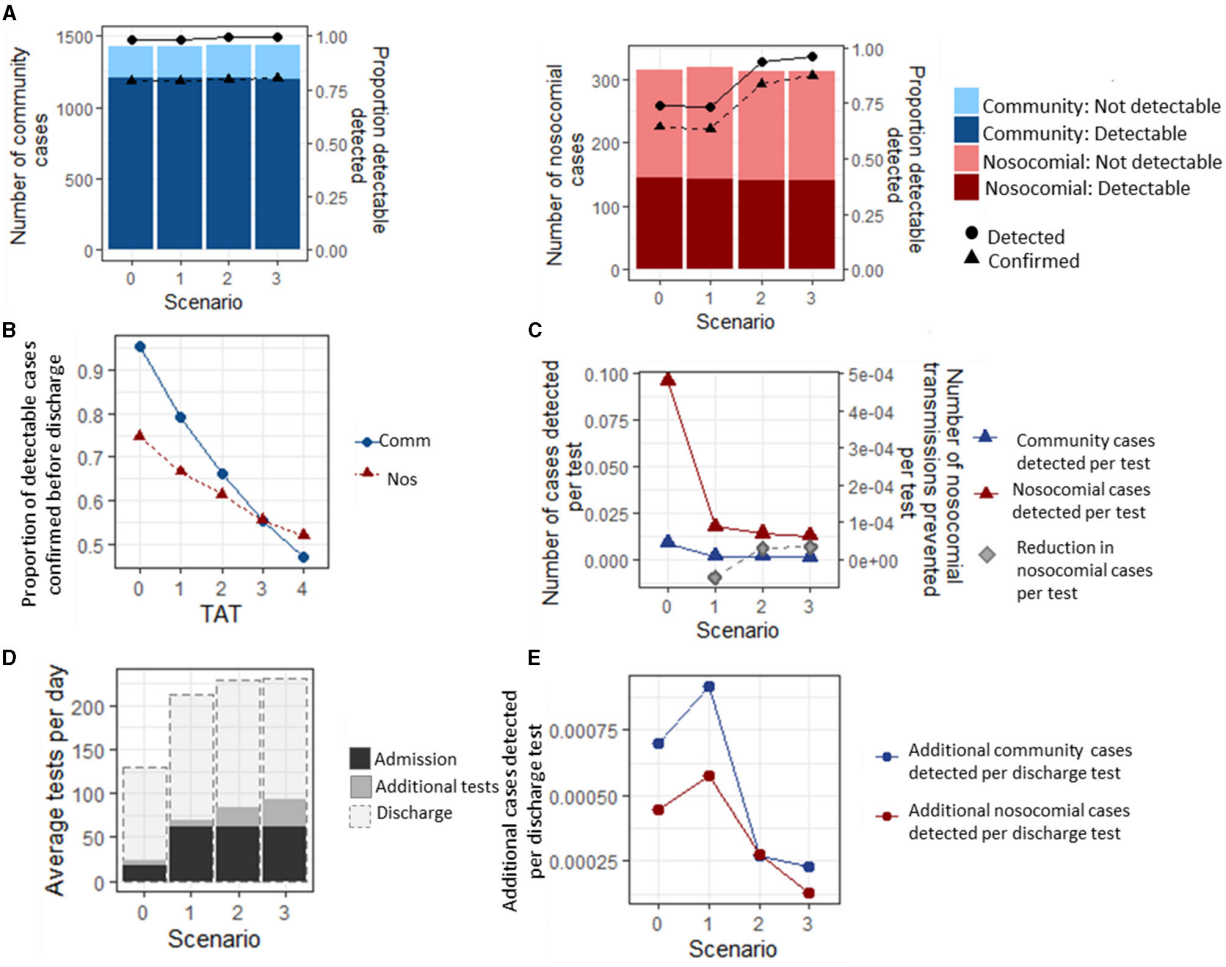
Infection status of community and nosocomially infected inpatients. **(A)** Number of community and nosocomial cases that are PCR detectable or not before discharge (bars), and proportion of detectable cases that are detected and confirmed before a patient is discharged (lines). **(B)** Average proportion of detected cases that have a confirmed result before discharge under different turnaround times (TATs) for testing strategy 0. under all scenarios. **(C)** Number of cases detected per test under each scenario. **(D)** Average number of tests performed per day under each testing strategy. **(E)** Additional cases detected per additional discharge test under each scenario, and reduction in nosocomial cases per additional test performed.

We explored the efficacy and efficiency of four testing strategies (Supplementary Table 2) implemented in England during the pandemic for detecting community and nosocomial COVID-19 infections. Under the baseline assumptions (see Methods section), 88.5% (88.2, 88.7) of community-acquired cases were detectable before discharge, and 98.0% (97.9, 98.1) of these cases (86.3% overall) were detected under Strategy 0 (Figure 2A). This increased to a maximum of 99.8% (99.7, 99.9) under Strategy 3. Assuming a 1 day turnaround time for test results, 78.9% (78.6–79.2) of detectable and 69.8% (69.3, 70.2) of all community-acquired cases were confirmed (i.e., results had been returned) under the baseline scenario before a patient was discharged. As the turnaround time for test results increased from 1 to 4 days, the proportion of detectable community-acquired cases that were confirmed before a patient was discharged decreased to 48.5% (48.1, 48.9) (Figure 2B). Nosocomial cases were less likely to be detectable before a patient was discharged from the hospital, but detectable cases had a similar detection rate when compared to community-acquired cases (Figure 2A). Only 40.8% (40.3, 41.3) of all nosocomial cases were detectable before discharge, but of these cases, 74.0% (73.1,4.9) (30.8% overall) were detected under Strategy 0, rising to 96.4% (96.0, 96.6) under Strategy 3. For nosocomial cases, 64.3% (63.3, 65.3) of detectable cases were confirmed under Strategy 0, assuming a 1 day turnaround time for test results, increasing to 80.4% (80.0, 80.6) under Strategy 3. As the turnaround time for results increased from 1 to 4 days, the proportion of nosocomial cases that were confirmed before discharge decreased to 48.8% (47.3, 49.5) (Figure 2B). It is possible that the remainder of the detectable cases could be identified by discharge testing.

The most efficient strategy for case detection (where efficiency is defined as the maximum detection rate per test) was Strategy 0, with 0.28 (0.27, 0.29) community-acquired cases and 0.026 (0.025, 0.027) nosocomial cases detected per test performed over the entire simulation period (Figure 2C). However, Strategies 1–3 all prevented nosocomial infections compared to Strategy 0, with the highest reduction in the number of nosocomial transmissions per test being 0.000050 (0.000048, 0.000051) under Strategy 3.

Under the baseline assumptions, adding testing patients on discharge increased the average number of tests per day by a factor of 5 for Strategy 0 and a factor of 2.5 for Strategy 3 (Figure 2D). Discharge testing had the greatest benefit under Strategy 1, where an additional 0.00172 (0.00171, 0.00174) nosocomial and 0.00057 (0.00055, 0.00061) community-acquired cases were detected per additional test for discharge, and the smallest benefit was under Strategy 3, where 0.00069 (0.00067, 0.00070) and 0.00038 (0.00037, 0.00040) nosocomial and community-acquired cases were detected per test performed (Figure 2E).

### Factors influencing the proportion of inpatients that develop a nosocomial infection

**Figure 3:**
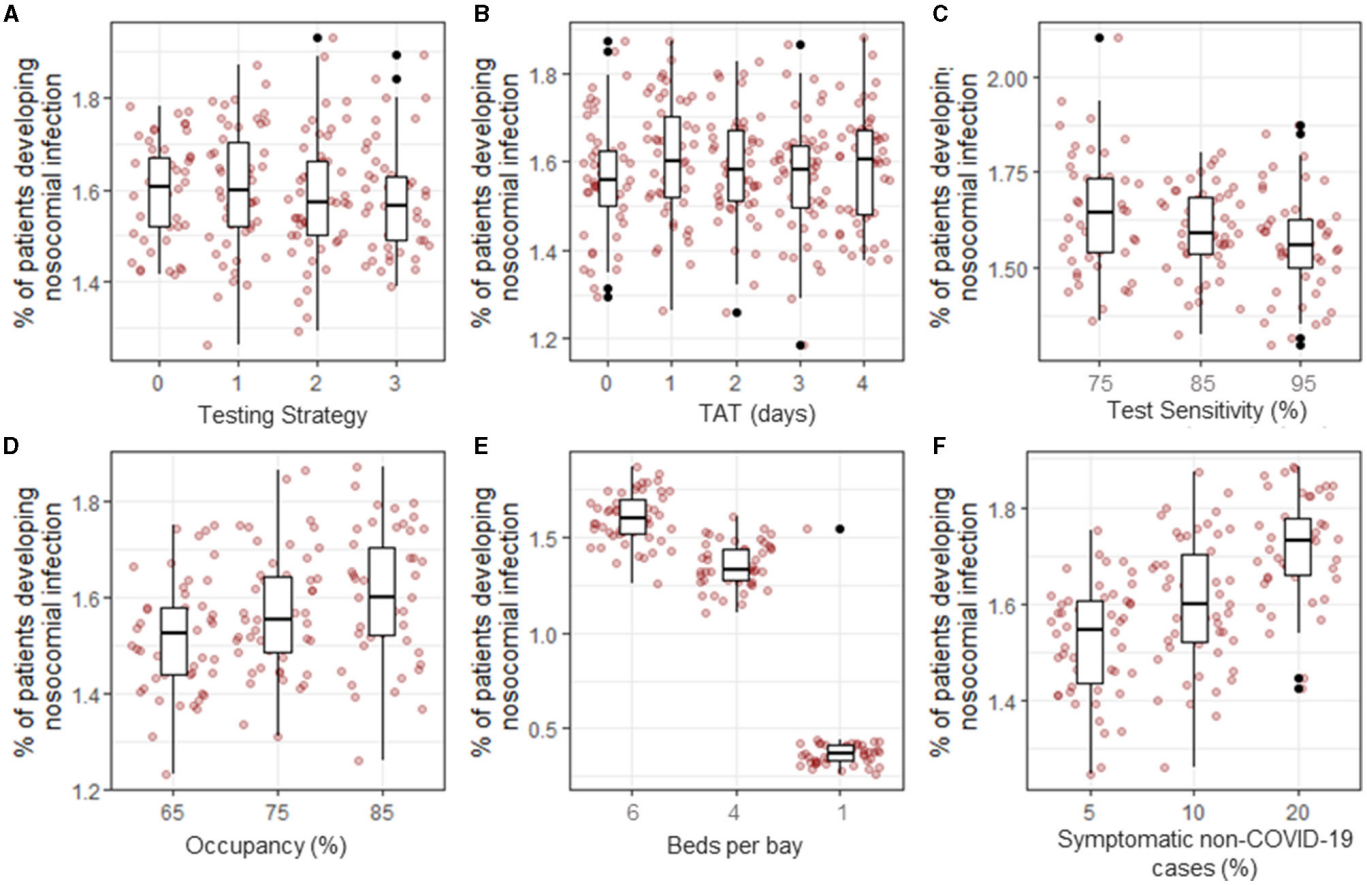
Proportion of inpatients that developed a nosocomial infection between 03-Mar-2020 and 01-Sept-2020 under different scenarios. The baseline parameter set (see *Methods*) was modified to explore the effect of testing strategy **(A)**, turnaround time **(**TAT, **B)**, test sensitivity **(C)**, hospital occupancy **(D)**, bay size **(E)**, and proportion of non-COVID-19 patients displaying COVID-19-like symptoms on admission **(F)**.

Despite there being a slight reduction in the number of nosocomial transmissions that occur between the different testing strategies (Figure 2C), there was not a significant difference in the overall proportion of patients who developed a nosocomial infection between the four testing strategies over the entire study period (1.61, 1.60, 1.58, and 1.56%, respectively, *p* = 0.76, Figure 3A). Similarly, there was no significant difference in the proportion of patients that developed a nosocomial infection when the turnaround time for test results increased from 1 to 4 days (1.56, 1.60, 1.59, 1.59, and 1.61% for TATs 0 to 4 days, respectively, *p* = 0.573, Figure 3B) or for rapid testing (0 day TAT), assuming the test specificity of both types of tests was 95%. If the test specificity were to decrease to either 85 or 75%, then the proportion of patients that developed a nosocomial infection would increase from 1.59 to 1.60 or 1.62%, respectively (*p* = 0.0129, Figure 3C).

Under the baseline assumptions, hospital occupancy was set to 85% to reflect the standard levels of occupancy in NHSE hospitals. The effect of reducing occupancy rates to 75% or 65%, as observed early in the first wave of the pandemic, was that the proportion of patients developing a nosocomial infection decreased from 1.59% (1.57, 1.62) to 1.57% (1.54, 1.61) or 1.51% (1.48, 1.55), respectively (*p* = 0.003, Figure 3D). However, these differences are very small and were not observable in patients admitted in any single week or time period (Supplementary Figure 3).

Reducing the number of beds in a bay from 6 to 4 resulted in a reduction of 0.2% in the proportion of patients that developed a nosocomial infection [from 1.59% (1.57, 1.62) to 1.35% (1.33, 1.39)], and if a hospital had the capacity to keep all patients in individual rooms or bays, the proportion of patients that develop a nosocomial infection declined to 0.38% (0.34, 0.44) (*p* < 0.001, Figure 3E). This difference was accompanied by a reduction in the percentage of patients that share a room with infected patients in a single day from 14.0% (13.9, 14.2) to 9.6% (9.5, 9.7) and 0%, respectively (Supplementary Figure 3), with the most strongly affected patients admitted between July and September, when nosocomially infected patients make up the highest proportion of infected patients in hospitals (Figure 1A). Patients with COVID-19-like symptoms are cohorted together on admission pending testing results. Changing the proportion of patients displaying COVID-19-like symptoms that are not genuine COVID-19 cases from 10% at baseline to 5% or 20% caused the overall proportion of patients that experienced a nosocomial infection to change from 1.59% (1.57, 1.62) to 1.52 (1.49, 1.55) or 1.71% (1.68, 1.75), respectively (*p* < 0.001, Figure 3F). This increase was observable throughout most of the simulation period but was most pronounced between May and June when the number of COVID-19 cases admitted from the community was highest (Figure 1A). Furthermore, as the proportion of patients that are wrongly suspected of having COVID-19 on admission increased, the proportion of patients that share a bay with non-infected patients in a single day also notably changed from 11.8% (11.7, 12.0) at 5% wrongly suspected to 17.3 (17.2,17.5) at 20% wrongly suspected (Supplementary Figure 3) under the baseline assumption that the hospital is laid out in six-bed bays and infectious patients could transmit to other patients in the same bay.

## Discussion

Using data-driven modeling, we estimated the proportion of SARS-CoV-2 infections among hospital inpatients that were detectable/infectious based on their length of stay. Our results suggest that patients who develop a nosocomial infection but have a shorter stay than 5 days are not infectious during their inpatient stay but will go on to become infectious later and can potentially contribute to onward transmission in the community. The relative contribution of such individuals to community transmission will likely be lowest when COVID-19 prevalence is high. Patients who develop a nosocomial infection with a length of stay of 5 to 25 days are highly likely to be infectious in hospital and would be detectable on discharge but were unlikely to be tested in hospital in the period under study due to being in an asymptomatic, pre-symptomatic, or very newly symptomatic state. This was also demonstrated in a study of long-term care facilities that suggested mild symptoms did not occur until 9 days after an interaction with an index case (42). As stated previously, the impact of discharge testing on return to social care facilities is not considered; however, we predict that had all patients been tested on discharge, a maximum of 26.0% of additional nosocomial infections would be identified under scenarios where patients were tested on admission. Similar results were obtained in a study using a different model, considering the impact of discharge testing on symptomatic case identification, which suggests that ~3% of all cases over the first wave may be linked to nosocomial transmission or transmission from discharged nosocomial cases that were not detected in the hospital (35). It should be noted, however, that in the first wave of the pandemic, there were considerable constraints on the availability of testing, and so it remains unknown what the true proportion of additional cases would have been if discharge testing had been practicable.

Community-acquired cases are only infectious for a short time post-discharge because they typically spend only a proportion of their infectious period outside the hospital. They have also been shown to contribute to fewer new cases than their nosocomial counterparts (35). Testing community-acquired cases on discharge would lead to an additional 2% of cases being identified. Discharge testing is most efficient when used in combination with a testing strategy where every patient is tested on admission, resulting in 0.00172 (0.00171, 0.00174) nosocomial and 0.00057 (0.00055, 0.00061) community-acquired cases detected per discharge test performed (Figure 2).

Over the first wave in England, 15,564 confirmed SARS-CoV-2 infections were detected in hospitalized individuals (14). Under our estimate that only 30.8% of all nosocomial cases were identified in hospitals before discharge, a result that is supported by other models from the same time period (35, 43), we would expect there to be an additional 34,968 cases that were missed. However, national data linking patient records to positive test results in the community identified only 14,913 cases in recently hospitalized individuals (14). This suggests that 39.7% of nosocomial cases were never identified, and the contribution of these cases to the transmission rate of SARS-CoV-2 in the community is unknown, although they could make up 4% of all new hospital admissions when readmitted (35). Repeat testing between days 5 and 7 alone or in addition to day 3 testing increases the proportion of detected nosocomial cases but does not significantly reduce nosocomial transmission rates in hospitals. This differs from closed settings such as long-term care facilities, where frequent testing has been shown to be more effective (44, 45).

We estimate that when only symptomatic patients are tested on admission, 26.0% of the 40.8% of nosocomial infections that are detectable before a patient is discharged from the hospital, and 2% of the 86.6% of community-acquired cases are missed (3.3% of all SARS-CoV-2 infections among hospital inpatients). These results are in line with other modeling studies from the same time period that estimate the proportion of nosocomial cases detected (35, 43) and also agree with studies estimating detection rates of SARS-CoV-2 in travelers entering the UK (46, 47). This strategy was in place in England up until 27 April 2020 (while testing availability was very constrained and the possibility of asymptomatic transmission was uncertain). During that time, over 53,000 patients were admitted to the hospital with a detected SARS-CoV-2 infection, and a further 17,000 had a SARS-CoV-2 infection that was potentially nosocomial in origin [positive test at least 3 days post-admission or within 14 days of discharge from the hospital (14)]. Over this time period, we estimate that in our simulated 1,000-bed hospital, 58 additional cases would have been detected by discharge testing over the first wave, equating to almost 6,000 nationally. Identifying these cases could have potentially reduced the burden of SARS-CoV-2 infections in previously hospitalized inpatients in the general community through reduced onward transmission.

We also explored the factors that could potentially impact nosocomial transmission rates in English hospitals. Correctly cohorting patients had the strongest effect on reducing nosocomial transmission rates. The effect of correctly cohorting patients was most strongly observed in scenarios where there was a smaller number of beds per bay (and therefore more bays available for keeping infected and non-infected patients separate) or where the proportion of patients with COVID-19-like symptoms that were SARS-CoV-2 negative on admission (and therefore wrongly cohort with true COVID-19 patients while awaiting test results) was smallest (so there was less chance of incorrectly cohorting susceptible patients with infected patients on admission).

This study has a number of limitations. First, patients are classified as detected if they are ever tested during the infection period. Symptomatic patients undergo probabilistic testing upon symptom development. This means that patients may be tested at the time of discharge and therefore be classified as detected (but not confirmed) when, in reality, they would be unlikely to have been given a test. Furthermore, the spatial arrangement of wards, shared spaces such as corridors and bathroom facilities, and their distribution across several buildings within a trust are not explicitly represented in the model. The model is calibrated to national data, but the testing strategies were held constant within a scenario, whereas in reality, guidelines for testing patients varied over time, and patients were cohorted differently between trusts as capacity allowed. In addition, we have assumed throughout this analysis that patients who are tested get their results regardless of whether they are discharged. If this were not the case, the number of detected cases would be up to 19.6% lower for community-acquired cases and 13.4% lower for nosocomial cases. As previously mentioned, constraints on testing availability, especially in the first few months of the pandemic, also reduced the proportion of detected cases and, in doing so, the potential for cross-infection. Despite these limitations, we believe that the results presented here are a good representation of what may have happened under different scenarios in England and provide evidence for public health strategies that can be applied outside of the English setting.

This study has important implications for infection prevention in hospitals and can be applied to contexts other than SARS-CoV-2. These results are particularly relevant as testing programmes for SARS-CoV-2 are scaled back across the NHS as well as if a new vaccine escape variant emerges. The study focuses on English hospitals, but the results are broadly applicable to other secondary-care systems where the rates and types of interactions within and between populations of patients and HCWs are similar.

## Conclusion

Between 03 March 2020 and 01 August 2020, over 30,000 people could have been discharged from hospitals in England while harboring a non-detected SARS-CoV-2 infection, only 3.3% of which would have been identified by discharge screening. In addition, 59.2% of nosocomial infections are not detectable or infectious before discharge and will become detectable and infectious post-discharge, potentially contributing to onward transmission in the community. However, the impact is likely to be small when community prevalence is high. The most efficient way to detect community and nosocomial cases is by testing all patients on admission and subsequent discharge. Retesting patients 3 days post-admission increases the identification rate of detectable nosocomial cases; however, these cases would be incorrectly attributed as community-acquired cases under current ECDC healthcare-associated case definitions (41). Increasing hospital occupancy, a higher number of beds per bay, and incorrectly cohorting patients with COVID-19-like symptoms on admission are associated with higher rates of nosocomial transmission.

## Data availability statement

The raw data supporting the conclusions of this article will be made available by the authors, without undue reservation.

## Author contributions

SE developed the model, performed the analysis, and wrote the article. JR contributed to the conception and design of the study. MW provided clinical insight and feedback on results. JS and DP provided data for model development. RH acquired and interpreted the underlying data. All authors contributed to the article and approved the submitted version.
