# Supplementary material for: Evaluating the impact of testing strategies for the detection of nosocomial COVID-19 in English hospitals through data-driven modeling
Source: Front Med (Lausanne). 2023 Oct 11;10:1166074. doi: 10.3389/fmed.2023.1166074 (PMC10622791; doi:10.3389/fmed.2023.1166074)
Supplement: Supplementary file 1 [file Table_1.DOCX]

**Table S1: Parameter values**

| **Parameter** | **Definition** | **Value** | **Source** |
| --- | --- | --- | --- |
| **General** |  |  |  |
| beds | Number of beds in trust | 1000 | Mean from individual trust data |
| bedsAvailable | Proportion of beds available for occupancy | 0.85 | Dependant on scenario |
| hcws | Number of HCWs | 8000 | Mean from individual trust data |
| patients | Number of patients initially in trust | 860 | 86% capacity from [Nuffield trust](https://www.nuffieldtrust.org.uk/resource/hospital-bed-occupancy#:~:text=Between%20Q1%202010%2F11%20and,%25%20in%20Q4%202019%2F20.) data |
| testSens | Sensitivity of test (false negative rate) | 0.90 | Woloshin et al, (9) |
| testSpec | Specificity of test (false positive rate) | 0.95 | Woloshin et al, (9) |
| incubationPeriodDays_shape | Shape variable for Gamma distribution for incubation period | 13.3 | He et al, (10) |
| incubationPeriodDays_rate | Rate variable for Gamma distribution for incubation period | 4.16 | He et al, (10) |
| onsetPeriodDays_mean | Mean for Log-normal distribution for onset period | 1.434065 | SPI-M communication |
| onsetPeriodDays_sd | SD for Log-normal distribution for onset period | 0.6612 | SPI-M Communication |
| camPop | Community population (Cambridgeshire) | 651482 | 2018 census |
| sharedRooms | Number of shared rooms available | 134 |  |
| singleRooms | Number of single rooms available | 196 | Fingertips (~20% beds are in single rooms) |
| bedsPerRoom | Number of beds in a shared room | 6 |  |
| **Patients** |  |  |  |
| probMale | Probability of patients being Male | 0.576 | SUS |
| ageShape | Shape component of Gamma distribution of age distribution | 4.145 | SUS |
| ageScale | Scale component of Gamma distribution of age distribution | 75.48 | SUS |
| shapeLOS_Susc | Shape parameter for LOS distribution Weibull distribution for susceptible patients | -0.1780082 | SUS |
| scaleLOS_Susc_int | Intercept for scale parameter for LOS distribution Weibull distribution for susceptible patients | 2.331949 | SUS |
| scaleLOS_Susc_age | Age component of scale parameter for LOS distribution Weibull distribution for susceptible patients | -0.00546131 | SUS |
| scaleLOS_Susc_female | Female gender component of scale parameter for LOS distribution Weibull distribution for susceptible patients | -1.242609 | SUS |
| scaleLOS_Susc_age_female | Interaction of age and female gender component of scale parameter for LOS distribution Weibull distribution for susceptible patients | 0.01424664 | SUS |
| shapeLOS_Inf | Shape parameter for LOS distribution Weibull distribution for symptomatically infected patients | 0.07853536 | SUS |
| scaleLOS_Inf_int | Intercept for scale parameter for LOS distribution Weibull distribution for symptomatically infected patients | 2.595361 | SUS |
| scaleLOS_Inf_age | Age component of scale parameter for LOS distribution Weibull distribution for susceptible patients | 0.002301093 | SUS |
| scaleLOS_Inf_female | Female gender component of scale parameter for LOS distribution Weibull distribution for susceptible patients | -0.5465226 | SUS |
| scaleLOS_Inf_age_female | Interaction of age and female gender component of scale parameter for LOS distribution Weibull distribution for susceptible patients | 0.006978023 | SUS |
| patientAsymProb | Probability a patient infection is asymptomatic | 0.4 | SUS |
| bP2P | Transmission rate to other patients | 0.000125 | Calibrated |
| bH2P | Transmission rate from HCW to patients | 0.0000001 | Calibrated |
| bP2P_hosp | Indirect transmission rate from patients to other patients |  | Calibrated |
| recoveryRatePat_Shape | Shape parameter of Gamma distribution of recovery distribution per day. | 1.43 | Fit to data from SUS |
| recoveryRatePat_Rate | Rate parameter of Gamma distribution of recovery distribution per day. | 0.542 | Fit to data from SUS |
| deathOnDisch_Inf_Int | Intercept of linear model for probability of death on discharge for infected patients | -0.00043572 | Fit to data from SUS |
| deathOnDisch_Inf_X | X component of linear model for probability of death on discharge for infected patients | -0.00159802 | Fit to data from SUS |
| deathOnDisch_Inf_X2 | X^2^ component of linear model for probability of death on discharge for infected patients | 7.30529E-05 | Fit to data from SUS |
| deathOnDisch_Susc_Int | Intercept of linear model for probability of death on discharge for non-symptomatic/uninfected patients | -7.96613 | Fit to data from Cohen et al. (7) |
| deathOnDisch_Susc_X | X component of linear model for probability of death on discharge for non-symptomatic/uninfected patients | 0.00102 | Fit to data from Cohen et al. (7) |
| deathOnDisch_Susc_X2 | X^2^ component of linear model for probability of death on discharge for non-symptomatic/uninfected patients | 0.000896 | Fit to data from Cohen et al. (7) |
| testOnAdmProb_Inf | Probability a symptomatically infected patient will be tested on admission | 0.95 | Estimated from individual NHSE trust data |
| testOnAdmProb_Other | Probability non-infected patient will be tested at random on admission | 0.05 | Estimated from individual NHSE trust data |
| testInHospProb | Probability a symptomatically infected patient infected nosocomially will be tested per timestep | 0.1 | Assumption that all patients will be tested within 2 days of developing symptoms |
| testPeriodSteps | Timesteps from test to result | 8 | Estimated from individual NHSE trust data |
| readmitProb | Probability a patient that develops symptoms after discharge will be readmitted within 14 days | 0.2 | Estimated from individual NHSE trust data |
| expOnAdmissionProb | Probability a susceptible patient will be exposed on admission (to be multiplied by the number of cases admitted that are known symptomatic) | 0.002631579 |  |
| retestProb_per_step | Probability a patient will be retested after day 5 (per step) |  | Dependent on scenario |
| hcw_to_patient_contacts | Number of unique patients seen by a HCW every day (used to calculate exposure risk to COVID+ patients) | 20 | (11) |
| non-covid_sympt_prob | Probability a patient admitted to hospital will have COVID-19 like symptoms but be SARS-CoV-2 negative | 0.05 | Dependent on scenario |
| **HCWs** |  |  |  |
| shiftLengthHrs | Length of shift in hours (this is converted to timesteps within the model code) | 12 | Assumption |
| hcwAsymProb | Probability a HCW that becomes infected is asymptomatic | 0.4 | Assumption |
| bP2H | Transmission probability from patients to HCWs per timestep | 0.0000025 | Calibrated |
| bH2H | Transmission probability from HCWs to other HCWs per timestep | 0.0000001 | Calibrated |
| absentThroughSick_self | Probability a HCW will self-isolate per timestep | 0.01 | 34% over total infected time (12). |
| testProbHCWDays | Periodicity of HCW testing. Default -1 = no testing | -1 |  |
| absentDays | Number of days to be off work following a positive test | 7 |  |
| beginTestingDay | Number of days after which to begin testing HCWs | 0 |  |
| commScale | Scale of community acquisition rate for HCWs | 0.052 | T&T data |
| patient_to_hcw_contacts | Number of HCWs seen by a patient per day (for scaling transmission risk) | 18 | (11) |
| **Comm** |  |  |  |
| careHomeProb | Probability a patient is from a care home | 0.02 | Health foundation communication |
| caseScale |  |  |  |
|  | Scaling factor for admissions rate | 1 |  |
