# Supplementary material for: Evaluating the impact of testing strategies for the detection of nosocomial COVID-19 in English hospitals through data-driven modeling
Source: Front Med (Lausanne). 2023 Oct 11;10:1166074. doi: 10.3389/fmed.2023.1166074 (PMC10622791; doi:10.3389/fmed.2023.1166074)
Supplement: Supplementary file 2 [file Table_2.docx]

|  |  | Day of inpatient stay | | | | | | |
| --- | --- | --- | --- | --- | --- | --- | --- | --- |
|  |  | 1 | 2 | 3 | 4 | 5 | 6 | 7 |
| Testing strategy | 0 | ○ |  |  |  |  |  |  |
|  | 1 | ○ ● |  |  |  |  |  |  |
|  | 2 | ○ ● |  |  |  | ● | ● | ● |
|  | 3 | ○ ● |  | ● |  | ● | ● | ● |

**Table S2: Testing strategy definitions.** Points indicate when patients are routinely tested with hollow circles representative of symptomatic cases and filled circles all patients. Blue indicates cases classified as community-onset and coral are nosocomial cases. In addition to days indicated newly symptomatic cases are tested as symptoms develop.
