# Supplementary figures and images for: Evaluating the impact of testing strategies for the detection of nosocomial COVID-19 in English hospitals through data-driven modeling
Source: Front Med (Lausanne). 2023 Oct 11;10:1166074. doi: 10.3389/fmed.2023.1166074 (PMC10622791; doi:10.3389/fmed.2023.1166074)

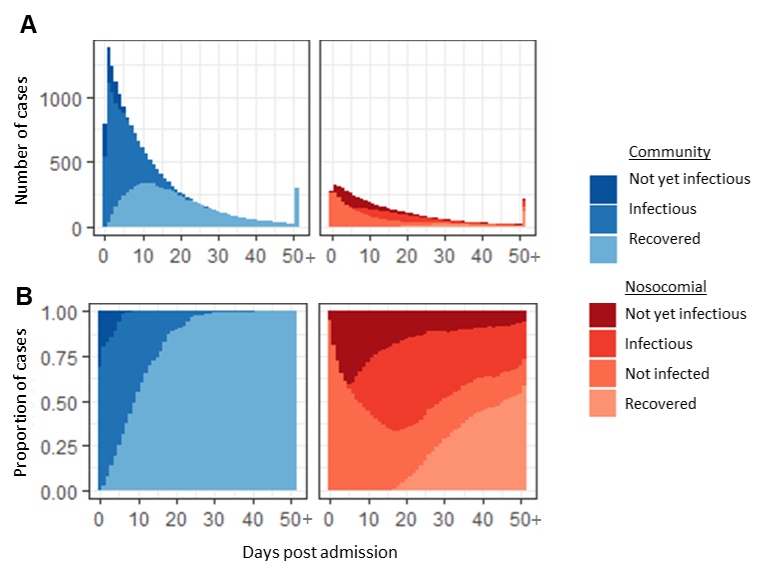

Supplement: Supplementary file 3 [file Image_1.tif]

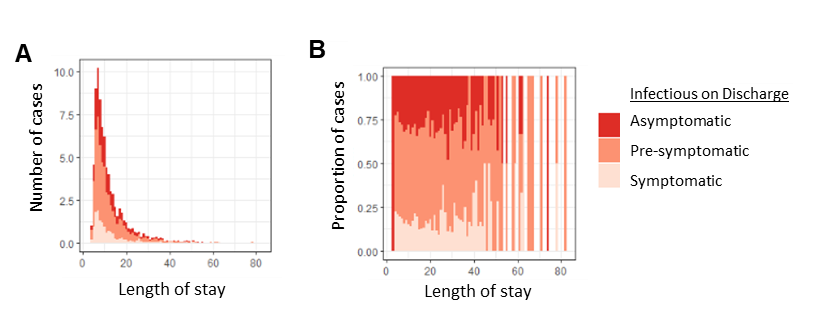

Supplement: Supplementary file 4 [file Image_2.tif]

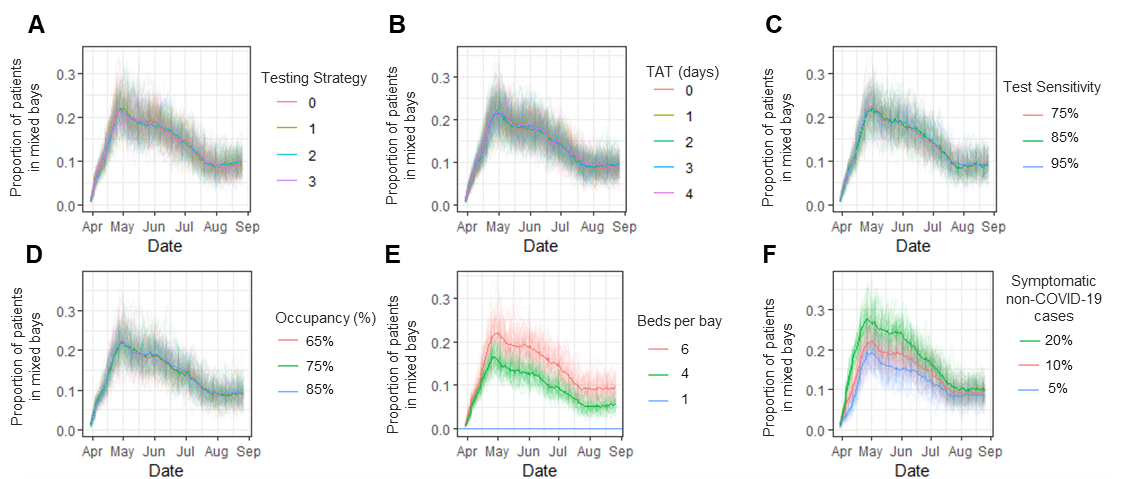

Supplement: Supplementary file 5 [file Image_3.tif]
